# Correlates of intimate partner violence among married women in Uganda: a cross-sectional survey

**DOI:** 10.1186/s12889-020-09123-4

**Published:** 2020-06-26

**Authors:** Derrick Gubi, Elizabeth Nansubuga, Stephen Ojiambo Wandera

**Affiliations:** 1 Department of Population Studies, School of Statistics and Planning, College of Business and Management Sciences, Makerere University, Kampala, Uganda; 2 Department of Demography & Population Studies, University of Witwatersrand, Johannesburg, South Africa

**Keywords:** Intimate partner violence, Alcohol, Witnessing parental violence, Controlling behaviors, Uganda

## Abstract

**Background:**

In Uganda, just like in many sub-Saharan countries, studies on Intimate Partner Violence (IPV) among married women are limited. The aim of this paper was to determine the correlates of emotional, sexual, physical IPV and any form of IPV among married women in Uganda.

**Methods:**

The 2016 Uganda Demographic and Health Survey (UDHS) data was used, and a weighted sample of 6879 married women were selected from the Domestic Violence module. Frequency distributions were used to describe the characteristics of respondents. Chi-square tests were used to establish the association between IPV and the explanatory variables. Binary logistic regressions were used to establish the factors that were associated with IPV among married women in Uganda.

**Results:**

More than half (56%) of the married women experienced some form of IPV. Sexual IPV was the least prevalent (23%) and 4 in 10 women (41 and 40%) experienced physical and emotional IPV, respectively. Factors associated with all the different forms of IPV included, age, region, witnessing parental violence, partner’s controlling behaviors, duration of the relationship, and frequency of intoxication of the male partner.

**Conclusion:**

IPV among Ugandan married women is far too common. This calls for collective efforts to reduce IPV in Uganda by addressing excessive alcohol consumption, controlling behaviors, and lack of awareness of the issue. Interventions aimed at preventing perpetration and tolerance of violence in the home settings should be promoted.

## Background

Globally, gender-based violence (GBV), also referred to as domestic violence, has gained momentum as a social, health and human rights issue [1, 2]. Of all forms of GBV, intimate partner violence (IPV) is the most common form, which involves all physical, sexual, or psychological harms as well as controlling behaviors aggravated by a current or former partner [3, 4]. The incidence of IPV is more severe in women compared to men with approximately 30% of women worldwide reporting violence by an intimate partner at some point in their life [5–7]. In low and middle income countries (LMICs), the prevalence of IPV is higher, with about 37% among women age 15–69 years [8].

In Uganda, IPV is still the highest contributor to GBV with a prevalence level of 40% among ever married women in the 12 months preceding the 2016 Uganda Demographic and Health Survey (UDHS), a rate which surpasses that of the rest of the world (30%) [9, 10]. While IPV has generally declined among women over time, its prevalence remains unacceptably high among Ugandan women. It contributes greatly to morbidity and mortality and its consequences are dire, including unwanted pregnancies, sexually transmitted infections, miscarriages, unsafe abortions, stillbirths, premature labour, low birth weight, anxiety, depression, among others [10–13]. Additionally, national statistics reveal that more than half (51%) of the cases of violence go unreported in Uganda [9]. This is partly due to the tolerance or acceptance of violence, which is rooted in socio-cultural beliefs that men are unconditionally entitled to sex [9].

The risk factors for IPV are complex and multifaceted, which calls for sound theoretical frameworks [14, 15]. Therefore, the nested ecological framework theory [16–18] and the social learning theory [16, 19, 20] were used to conceptualize this study. Multi-level hierarchical relationships at the proximal, intermediate and distal levels are explored in relation to IPV [21].

Socio-demographic factors like age, education, region and wealth status have been associated with IPV [1, 13, 21–27]. These function through intermediate factors (partners’ behavioral factors, history of violence and marital factors) to drive IPV. Intermediate factors, especially controlling behaviors, precede and catalyse IPV [25, 28]. Also, the economic empowerment of women, which is assessed by a woman owning a house or land (either alone or jointly with a partner) or receiving cash payment for her work, is a predictor of IPV.

Some studies on IPV in Uganda have been done in consonance with Article 33 of the Constitution of the Republic of Uganda and the Domestic Violence Act 2010 [24–26, 29–31]. However, studies focusing on “all forms of IPV” are limited. In addition, marital factors remain under-investigated. Therefore, this study considered marital factors (duration of relationship, number of co-wives, age at first marriage and parity) in its conceptualization. This study sought to establish the correlates of all forms of IPV among married women in Uganda.

## Methods

### Data source, study population and sample size

The study used data from the 2016 Uganda Demographic and Health Survey (UDHS), accessed with permission from DHS Program [32]. The UDHS (2016) was a cross-sectional nationally representative survey capturing national and sub-national estimates including, but not limited to, domestic violence and IPV in particular.

This survey employed a two stage stratified sampling procedure and cluster sampling design based on the sampling frame from the 2014 National Population and Housing Census [9, 33]. The domestic violence module, however, was based on the shortened and modified version of the Conflict Tactics Scale (CTS) [34]. An in-depth description of the sampling procedure is reported in the 2016 UDHS report [9].

This study used a sample of 9232 women selected in the domestic violence module who were age 15–49 years. From this sample, a weighted sample of 6879 married women (either married or cohabiting) were selected for the analyses. We used the domestic violence weighting variable (d005) included in the UDHS data and the Stata survey (svy) command to weight the data during the analyses in order to account for the complex survey design [35].

### Variables and measures

**Fig. 1:**
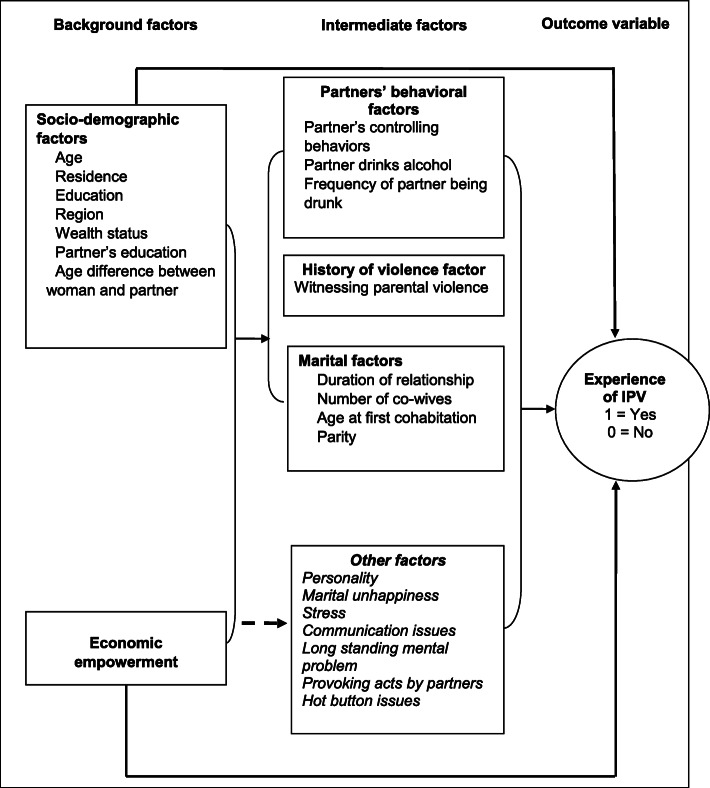
Conceptual framework (Adapted from Silva et al., 2015). The figure shows multiple pathways through which background and intermediate factors might drive intimate partner violence. Region and harmful use of alcohol are used as an example to illustrate the connectedness of background factors to intermediate factors in perpetuating IPV. Addiction to local beer (which is part of culture in some regions in Uganda) leads to drunkardness, which is a precursor to the experience of IPV. Therefore, region (a socio-demographic characteristic) can function through harmful use of alcohol (intermediate factor) to drive IPV

Figure 1 shows the conceptual framework used in selecting different sets of variables and measures used in the study. Categories of variables included outcome and explanatory variables.

### Outcome variables

The outcome variables were the three different forms of IPV (emotional, physical and sexual). In addition, an aggregate measure of IPV, which combined all the three forms of violence was generated. The dependent variables were based on the following set of questions asked to women in the survey. Women indicated whether their husbands/partners had ever or did;

- Hit, slap, kick or do anything else to hurt them physically?
- Force them to have intercourse or perform any other sexual acts against their will?
- Say something to humiliate them in front of others, threaten to hurt them or someone they care about, insult them or make them feel bad.

The response expected was either ‘yes’ or ‘no’; with ‘yes’ to the questions a, b, and c implying experience of physical, sexual and emotional IPV respectively and ‘no’ implying no experience of IPV. In addition, a ‘yes’ to any of the three questions a, b and c implied experience of any IPV and a ‘no’ implied no experience of any IPV.

### Measures of explanatory variables

The independent variables were classified into two broad categories, background factors and intermediate factors. Background factors included socio-demographic factors and economic empowerment, while intermediate factors included partners’ behavioral factors, history of witnessing parental violence and marital factors.

The socio-demographic characteristics included women’s age, place of residence, women’s education level, region, wealth status index, partner’s education, parity and age difference. Economic empowerment included female ownership of property (house and land) and type of earning from a woman’s work. It was obtained by merging women responses to questions: does a respondent: a) own a house? b) own land [either alone or jointly with a partner for both questions (a) and (b)] and c) the type of earning from her work. Analysis dichotomized question c into paid (cash only, cash and in kind, and in kind only) and not paid. A ‘yes’ to any of the three questions a, b and c implied that a woman was considered empowered and a ‘no’ implied non empowerment. Responses to these questions were recoded into two categories (0 = Not empowered, 1 = Empowered).

Partners’ behavioral factors comprised of partner’s controlling behaviors, partner’s alcohol consumption and frequency of a husband / partner being drunk. To measure the partner’s controlling behaviors, female participants were asked, “Does your partner ever or did; a) Prohibit you to meet female friends? b) Limit you contact your family? c) Insist on knowing where you are at all times? d) Is jealous if you talk with other men? and e) Frequently accuses you of being unfaithful?” These were merged into one variable called the “partner’s controlling behaviors.” Any affirmative response (yes) to any of the above questions implied presence of partner’s controlling behaviors and no to all the questions implied non-existence of such behaviors. The partner’s alcohol consumption was measured by responses to the question, “Does your partner drink alcohol?” and it had a binary outcome (0 = No, 1 = Yes). Frequency of a partner being drunk was a follow-up question to those respondents whose partners indicated that the partner drank alcohol.

History of violence comprised witnessing parental violence and was measured by whether the respondent’s father ever beat her mother. It had a binary outcome (0 = No, 1 = Yes). The study also considered marital factors which included duration of relationship, number of co-wives, parity and age at first cohabitation/marriage. Both number of co-wives and age at first marriage had binary outcomes, duration of relationship was categorised as 0 = 0–4 years, 1 = 5–9 years, 2 = 10–14, 3 = 15–19 and 4 = 20+ years. Parity was categorised as 0 = None, 1 = 1–4 and 2 = 5+. In this study, the term “partner” included husbands and also partners in cohabiting relationships.

### Statistical analyses

Data analysis was done using Stata version 14. Descriptive statistics (specifically frequency distributions) were used at univariate analysis to describe the characteristics of the respondents. Pearson chi-square tests were used to test initial associations. Finally, multivariable logistic regressions were used to assess the association of the explanatory factors on the experience of emotional, sexual, physical IPV and any form of IPV as an aggregate measure of the three variables. Also, goodness of fit of the different models was tested and checked by running the link test.

## Results

### Prevalence of IPV

Almost an equal number of women experienced both physical and emotional forms of IPV (41 and 40% respectively), while sexual IPV was the least common (23%). Overall, the majority (56%) of the women experienced any form of IPV.

### Descriptive characteristics of respondents

Table 1 presents the background characteristics of respondents. More than half (76%) of the women resided in rural areas, had primary education (60%), were economically empowered (86%), and their partners had mostly a primary education (53%). Fifty percent of women reported an age difference of 0–5 years with their partners. There was nearly an even distribution of women by region. The Northern region had the fewest (20%) women, while the Central region the most (28%) representation.

**Table 1:** Socio-demographic background characteristics of Respondents

Background characteristics	Frequency (n)	Percentage (%)
**Age**		
15–24	1951	28
25–34	2544	37
35+	2384	35
**Residence**		
Urban	1620	24
Rural	5259	76
**Respondent’s education**		
No education	891	13
Primary	4125	60
Secondary+	1864	27
**Partner’s education**		
No education	504	9
Primary	2990	53
Secondary+	2149	38
**Region**		
Central	1928	28
Eastern	1833	27
Northern	1384	20
Western	1734	25
**Wealth status**		
Poorest	1334	19
Poorer	1400	20
Middle	1349	20
Richer	1296	19
Richest	1500	22
**Parity**		
None	388	6
1–4	3796	55
5+	2695	39
**Age difference between woman and partner**		
Wife older	318	6
0–5 age gap	2793	50
6+ age gap	2531	45
**Economic empowerment status**		
Not empowered	976	14
Empowered	5903	86
**Total**	**6879**	**100**

Table 2 presents other IPV related factors. The majority (71%) of women experienced partner controlling behaviors, married after 18 years of age (52%), never had co-wives (74%) and had partners that never drank alcohol (57%). About 2 in 5 women witnessed parental violence (36%).

**Table 2:** Distribution of IPV related characteristics of the Respondents

Characteristics	Frequency (n)	Percentage (%)
**Witnessing parental violence**		
No	4403	64
Yes	2476	36
**Duration of relationship in years**		
0–4	1750	25
5–9	1444	21
10–14	1044	15
15–19	984	14
20+	1657	24
Number of co-wives*		
None	4187	74
One or more co-wives	1456	26
**Age at first marriage**		
Below 18 years	3299	48
18 or more years	3580	52
**Partner controlling behaviors**		
No	1965	29
Yes	4914	71
**Partner drinks alcohol**		
No	3906	57
Yes	2973	43
**Frequency of partner being drunk**		
Never gets drunk	4231	62
Often	1079	16
Sometimes	1569	23
**Total**	**6879**	**100**

* Frequencies do not add up to 6879 due to missing responses and/ or filters that dropped some questions when certain criterion was not met

### Association between IPV and explanatory variables

Table 3 presents cross tabulations for associations between the different explanatory variables and experience of the different IPV forms. Except for partner’s age differences, all other factors were significantly associated with either emotional, sexual, physical or any IPV. Remarkably, women who were over the age of 35, resided in a rural area, married before age 18, represent the poorest wealth quintile, or personally witnessed parental violence also experienced all forms of IPV. There was an almost even distribution of IPV by region. Women whose partners had controlling behaviors, consumed alcohol and had more than one wife experienced more IPV, while those whose partners had a secondary education or above had the least prevalence of IPV.

**Table 3:** Association between explanatory variables and IPV

Variables	Emotional IPV		Sexual IPV		Physical IPV		Any IPV		Total
	%	n	%	n	%	n	%	n	
**Age group**									
15–24	34.8	679	20.5	400	33.6	655	49.3	962	1951
25–34	40.6	1034	23.2	591	38.4	976	54.3	1383	2544
35+	46.6	1111	24.5	584	47.4	1131	62.7	1495	2384
**Residence**									
Urban	36.9	598	18.4	298	33.3	539	46.8	758	1620
Rural	42.3	2226	24.3	1278	42.3	2223	58.6	3081	5259
**Education**									
No education	47.3	422	21.3	190	47.6	424	61.5	548	891
Primary	44.3	1827	25.9	1066	44.5	1834	60.6	2500	4125
Secondary+	30.9	575	17.1	319	27.0	504	42.5	792	1864
**Partner’s education**									
No education	42.3	213	18.9	95	40.5	204	56.6	285	504
Primary	42.3	1264	24.4	730	43.5	1300	59.7	1784	2990
Secondary+	31.1	669	17.2	369	27.8	597	43.4	932	2149
**Region**									
Central	32.3	624	18.4	354	31.2	602	44.9	866	1928
Eastern	39.6	726	28.9	530	41.4	758	58.1	1065	1833
Northern	44.5	616	17.8	247	49.2	681	61.3	848	1384
Western	49.6	859	25.7	445	41.5	720	61.1	1060	1734
**Wealth status**									
Poorest	47.6	634	23.1	308	52.3	698	65.9	879	1334
poorer	42.5	595	26.0	365	44.9	629	59.8	837	1400
Middle	44.4	599	25.4	342	41.3	557	59.3	801	1349
Richer	40.7	528	25.0	324	37.2	482	55.5	719	1296
Richest	31.2	469	15.8	236	26.4	396	40.3	604	1500
**Age difference between woman and partner**									
Wife older	40.1	128	21.5	68	38.4	122	56.8	180	318
0–5 age gap	37.7	1054	21.9	611	38.0	1062	53.3	1490	2793
6+ age gap	38.1	964	20.4	515	36.2	917	52.6	1331	2531
**Economic empowerment status**									
Not empowered	34.8	339	23.7	232	35.5	346	52.3	510	979
Empowered	42.1	2485	22.8	1344	40.9	2416	56.4	3329	5903
**Partner controlling behaviors**									
No	16.4	322	7.6	149	19.6	384	28.9	567	1965
Yes	50.9	2502	29.0	1426	48.4	2377	66.6	3272	4914
**Partner drinks alcohol**									
No	31.7	1239	18.5	724	29.3	1146	45.3	1770	3906
Yes	53.3	1585	28.6	851	54.3	1616	69.6	2070	2973
**Frequency of partner being drunk**									
Never gets drunk	31.6	1337	18.4	777	29.3	1240	45.1	1906	4231
Often	70.4	760	37.7	406	74.5	804	83.8	905	1079
Sometimes	46.4	727	25.0	392	45.7	718	65.6	1028	1569
**Witnessing parental violence**									
No	35.1	1547	18.4	811	33.9	1494	48.9	2153	4403
Yes	51.6	1277	30.9	764	51.2	1267	68.1	1686	2476
**Duration of relationship in years**									
0–4								732	1750
5–9	42.1	607	24.8	358	40.5	585	55.9	807	1444
10–14	45.5	475	25.1	263	43.1	450	60.2	629	1044
15–19	43.9	431	24.7	243	42.6	419	58.7	578	984
20+	49.1	814	25.1	416	51.4	852	66.0	1094	1657
**Number of co-wives**									
None	36.1	1511	20.3	851	35.1	1471	51.1	2137	4187
One or more co-wives	43.5	643	23.4	344	43.3	630	59.4	864	1456
**Age at first marriage**									
Below 18 years	45.2	1490	23.3	770	45.8	1512	60.3	1991	3299
18+ years	37.3	1334	22.5	805	34.9	1250	51.7	1849	3580
**Parity**									
None	23.6	92	15.4	60	18.0	70	34.9	135	388
1–4	38.7	1471	21.1	802	37.0	1405	52.3	1987	3796
5+	46.8	1262	26.5	714	47.8	1287	63.7	1718	2695
**Total**	**41.1**	**2824**	**22.9**	**1575**	**40.2**	**2762**	**55.8**	**3839**	**6879**

### Multivariate results

Table 4 shows the net influence of explanatory variables on occurrence of emotional, sexual, physical and any IPV. For all the models, the variables that were not significant at the bivariate level of analysis were excluded.

**Table 4:** Results of logistic regressions of the different forms of IPV and the explanatory factors

Characteristics	*Model on emotional IPV*			*Model on sexual IPV*			*Model on physical IPV*			*Model on any IPV*		
	*OR*	*95% CI*	*p-value*	*OR*	*95% CI*	*p-value*	*OR*	*95% CI*	*p-value*	*OR*	*95% CI*	*p-value*
**Age group**												
15–24 (RC)	1.00			1.00			1.00			1.00		
25–34	0.79	(0.64–0.98)	**0.03**	0.78	(0.61–0.99)	**0.04**	0.76	(0.60–0.96)	**0.02**	0.75	(0.60–0.95)	**0.02**
35+	0.88	(0.61–1.28)	0.51	0.74	(0.50–1.10)	0.13	0.86	(0.59–1.26)	0.44	0.81	(0.55–1.19)	0.28
**Education**												
No education (RC)	1.00			1.00			1.00			1.00		
Primary	1.05	(0.84–1.32)	0.67	1.37	(1.07–1.76)	**0.01**	1.25	(0.99–1.58)	0.06	1.20	(0.97–1.49)	0.10
Secondary+	0.89	(0.67–1.19)	0.44	1.00	(0.73–1.36)	1.00	1.01	(0.76–1.36)	0.92	1.02	(0.78–1.33)	0.89
**Partner’s education**												
No education (RC)	1.00			1.00			1.00			1.00		
Primary	1.01	(0.78–1.13)	0.92	1.27	(0.97–1.67)	0.08	1.20	(0.95–1.52)	0.13	1.19	(0.93–1.52)	0.16
Secondary+	0.85	(0.64–1.13)	0.27	1.04	(0.77–1.40)	0.78	0.89	(0.68–1.17)	0.39	0.89	(0.67–1.19)	0.44
**Region**												
Central (RC)	1.00			1.00			1.00			1.00		
Eastern	1.08	(0.84–1.41)	0.54	1.53	(1.16–2.01)	**0.00**	1.04	(0.82–1.32)	0.75	1.18	(0.89–1.55)	0.25
Northern	1.31	(1.01–1.70)	**0.04**	0.80	(0.59–1.08)	0.14	1.31	(1.02–1.69)	**0.04**	1.28	(1.01–1.62)	**0.04**
Western	2.17	(1.75–2.70)	**0.00**	1.51	(1.18–1.93)	**0.00**	1.28	(1.02–1.62)	**0.04**	1.69	(1.37–2.08)	**0.00**
**Wealth status**												
Poorest (RC)	1.00			1.00			1.00			1.00		
Poorer	0.88	(0.70–1.10)	0.27	1.10	(0.86–1.40)	0.45	0.79	(0.63–1.00)	**0.04**	0.80	(0.64–1.01)	0.06
Middle	1.04	(0.78–1.38)	0.80	1.07	(0.82–1.41)	0.62	0.71	(0.56–0.91)	**0.01**	0.83	(0.65–1.07)	0.15
Richer	0.94	(0.72–1.23)	0.65	1.19	(0.89–1.59)	0.25	0.67	(0.51–0.89)	**0.01**	0.78	(0.61–1.00)	0.05
Richest	0.93	(0.67–1.30)	0.69	1.01	(0.69–1.47)	0.98	0.53	(0.39–0.72)	**0.00**	0.63	(0.47–0.84)	**0.00**
**Witnessing parental violence**												
No (RC)	1.00			1.00			1.00			1.00		
Yes	1.74	(1.48–2.05)	**0.00**	1.75	(1.49–2.06)	**0.00**	1.66	(1.42–1.94)	**0.00**	1.90	(1.62–2.23)	**0.00**
**Partner controlling behaviors**												
No (RC)	1.00			1.00			1.00			1.00		
Yes	5.24	(4.56–6.46)	**0.00**	4.19	(3.36–5.22)	**0.00**	3.66	(3.06–4.37)	**0.00**	5.20	(4.46–6.07)	**0.00**
**Economic empowerment**												
Not empowered (RC)	1.00			1.00			1.00			1.00		
Empowered	1.09	(0.88–1.35)	0.44	0.77	(0.60–0.99)	**0.04**	0.90	(0.73–1.12)	0.36	0.82	(0.66–1.02)	0.07
**Duration of relationship in years**												
0–4 (RC)	1.00			1.00			1.00			1.00		
5–9	1.75	(1.41–2.18)	**0.00**	1.66	(1.28–2.14)	**0.00**	1.85	(1.49–2.30)	**0.00**	1.85	(1.50–2.29)	**0.00**
10–14	2.11	(1.60–2.78)	**0.00**	1.87	(1.32–2.64)	**0.00**	2.12	(1.60–2.82)	**0.00**	2.52	(1.91–3.34)	**0.00**
15–19	1.84	(1.32–2.58)	**0.00**	1.92	(1.27–2.91)	**0.00**	1.97	(1.39–2.79)	**0.00**	2.24	(1.56–3.22)	**0.00**
20+	1.97	(1.28–3.03)	**0.00**	1.73	(1.05–2.85)	**0.03**	2.60	(1.70–3.97)	**0.00**	2.76	(1.75–4.35)	**0.00**
**Number of co-wives**												
None (RC)	1.00			1.00			1.00			1.00		
One or more co-wives	1.16	(0.98–1.37)	0.09	1.09	(0.90–1.31)	0.38	1.08	(0.91–1.28)	0.40	1.05	(0.89–1.25)	0.55
**Age at first marriage**												
Below 18 years (RC)	1.00			1.00			1.00			1.00		
18+ years	0.97	(0.83–1.14)	0.73	1.40	(1.16–1.69)	**0.00**	0.98	(0.83–1.15)	0.77	1.15	(0.99–1.35)	0.07
**Frequency of partner being drunk**												
Never (RC)	1.00			1.00			1.00			1.00		
Often	3.61	(2.48–5.25)	**0.00**	2.16	(1.43–3.25)	**0.00**	3.36	(2.48–5.31)	**0.00**	4.19	(2.87–6.12)	**0.00**
Sometimes	1.91	(1.34–2.73)	**0.00**	1.42	(0.95–2.12)	0.09	1.59	(1.13–2.24)	**0.01**	2.51	(1.79–3.54)	**0.00**
**Parity**												
None (RC)	1.00			1.00			1.00			1.00		
1–4	1.54	(1.09–2.18)	**0.02**	1.21	(0.80–1.81)	0.34	2.22	(1.59–3.08)	**0.00**	1.76	(1.27–2.44)	**0.00**
5+	1.61	(1.07–2.43)	**0.02**	1.49	(0.95–2.35)	0.09	2.28	(1.56–3.33)	**0.00**	1.97	(1.35–2.89)	**0.00**

Age had a significant relationship with all forms of IPV. Women age 25–34 years had lower odds of experiencing emotional IPV (OR = 0.79; 95% CI: 0.64–0.98), sexual IPV (OR = 0.78; 95% CI: 0.61–0.99), physical IPV (OR = 0.76; 95% CI: 0.60–0.96) and any IPV (OR = 0.75; 95% CI: 0.60–0.95) compared to those age 15–24 years.

Women’s education was associated with sexual IPV only. Women with primary education had higher odds (OR = 1.37; 95% CI: 1.07–1.76) of experiencing sexual violence compared to those with no education.

Region was related to all the forms of IPV. In particular, the odds of experiencing emotional IPV were higher among women in the Northern (OR = 1.31; 95% CI: 1.01–1.70) and Western regions of Uganda (OR = 2.17; 95% CI: 1.75–2.70). The odds of sexual IPV were higher in the Eastern (OR = 1.53; 95% CI: 1.16–2.01) and Western (OR = 1.51; 95% CI: 1.18–1.93) regions of Uganda than in the Central region. Like emotional IPV, the odds of physical IPV were higher in the Northern (OR = 1.31; 95% CI: 1.02–1.69) and Western regions of Uganda (OR = 1.28; 95% CI: 1.02–1.62). Overall, the odds of experiencing any IPV were higher among women in the Northern (OR = 1.28; 95% CI: 1.01–1.62) and Western (OR = 1.69; 95% CI: 1.37–2.08) regions of Uganda compared to the Central region.

Wealth status was strongly associated with physical IPV and any IPV. Specifically, women in the poorer, middle, richer and richest wealth quintiles had lower odds (OR = 0.79; 95% CI: 0.63–1.00, OR = 0.71; 95% CI: 0.56–0.91, OR = 0.67; 95% CI: 0.51–0.89 and OR = 0.53; 95% CI: 0.39–0.72 respectively) of experiencing physical IPV compared to those from the poorest wealth quintile.

The frequency of a husband/ partner being drunk was related to all forms of IPV. Women whose partners were “often” drunk had higher odds of experiencing emotional violence (OR = 3.61; 95% CI: 2.48–5.25), sexual violence (OR = 2.16; 95% CI: 1.43–3.25), physical violence (OR = 3.36; 95% CI: 2.48–5.31) and any IPV (OR = 4.19; 95% CI: 2.87–6.12), compared to those whose partners were never drunk. Similarly, women whose partners were “sometimes” drunk were more likely to experience emotional IPV (OR = 1.91; 95% CI: 1.34–2.73), physical IPV (OR = 1.59; 95% CI: 1.13–2.24) and any IPV (OR = 2.51; 95% CI: 1.79–3.54).

Partners’ controlling behaviors were strongly associated with all forms of IPV. Women who reported partners’ controlling behaviors had higher odds of experiencing emotional IPV (OR = 5.24; 95% CI: 4.56–6.46), sexual IPV (OR = 4.19; 95% CI: 3.36–5.22), physical IPV (OR = 3.36; 95% CI: 3.06–4.37) and any form of IPV (OR = 5.20; 95% CI: 4.46–6.07).

Furthermore, witnessing parental violence was associated with IPV. All women who witnessed parental violence had higher odds of experiencing emotional IPV (OR = 1.74; 95% CI: 1.48–2.05), sexual IPV (OR = 1.75; 95% CI: 1.49–2.06), physical IPV (OR = 1.66; 95% CI: 1.42–1.94 and any IPV (OR = 1.90; 95% CI: 1.62–2.23) compared to those who did not.

Duration of relationship had a significant association with all forms of IPV. The odds of experiencing emotional IPV were higher among women with 5–9 years’ marital duration (OR = 1.75; 95% CI: 1.41–2.18), 10–14 years’ duration (OR = 2.11; 95% CI: 1.60–2.78), 15–19 years’ duration (OR = 1.84; 95% CI: 1.32–2.58) and 20+ years’ duration (OR = 1.97; 95% CI: 1.28–3.03). For sexual IPV, the odds were higher for women with 5–9 years’ duration (OR = 1.66; 95% CI: 1.28–2.14), 10–14 years’ duration (OR = 1.87; 95% CI: 1.32–2.64), 15–19 years’ duration (OR = 1.92; 95% CI: 1.27–2.91) and 20+ years’ duration (OR = 1.73; 95% CI: 1.05–2.85). The odds of experiencing physical IPV were higher for women with 5–9 years’ duration (OR = 1.85; 95% CI: 1.49–2.30), 10–14 years’ duration (OR = 2.12; 95% CI: 1.60–2.82), 15–19 years’ duration (OR = 1.97; 95% CI: 1.39–2.79) and 20+ years’ duration (OR = 2.60; 95% CI: 1.70–3.97). The odds of experiencing any IPV were higher for women with 5–9 years’ duration (OR = 1.85; 95% CI: 1.50–2.29), 10–14 years’ duration (OR = 2.52; 95% CI: 1.91–3.34), 15–19 years’ duration (OR = 2.24; 95% CI: 1.56–3.22) and 20+ years’ duration (OR = 2.76; 95% CI: 1.75–4.35).

Age at first marriage was related with sexual IPV only. Women who married after 18 years were more likely (OR = 1.40; 95% CI: 1.16–1.69) to experience sexual violence compared to those who married before the age of 18.

Economic empowerment showed a significant association with sexual IPV. Women who were empowered had lower odds (OR = 0.77; 95% CI: 0.60–0.99) of experiencing sexual IPV compared to those who were not empowered.

Parity was the other predictor of IPV. Women with parity of 1–4 children had higher odds of experiencing emotional IPV (OR = 1.54; 95% CI: 1.09–2.18), physical IPV (OR = 2.22; 95% CI: 1.59–3.08) and any IPV (OR = 1.76; 95% CI: 1.27–2.44). Similarly, women with 5 children and more, were also more likely to experience emotional violence (OR = 1.61; 95% CI: 1.07–2.43), physical violence (OR = 2.28; 95% CI: 1.56–3.33) and general violence (OR = 1.97; 95% CI: 1.35–2.89).

## Discussion

The aim of this study was to establish the correlates of IPV among married women in Uganda. The prevalence of IPV among these women remains relatively high (56%) compared to an average of 37% for other LMICs [8]. From the results, woman’s education, region, wealth status, age, witnessing parental violence, partner controlling behaviors, duration in relationship, frequency of partner being drunk, age at first marriage and parity were significantly associated with IPV in various ways.

Women’s education was significantly associated with sexual IPV as women with primary education had increased odds of experiencing sexual violence compared to those with no education. A similar finding was reported in Ethiopia [36], India [37], South Africa [38] and the WHO multi-country study [13]. Perhaps women with no education are more submissive to their partners and less likely to face IPV compared to those with some education. Also, it could be that women with a primary education are more empowered to recognize and report (during the survey) their experience of IPV as compared to those with no education. While the partner’s education was a significant predictor of domestic violence in the WHO study [13], it did not predict IPV against married women (either married or cohabiting) in Uganda. It is possible that mass media “edutainment” strategies have not yet changed social norms or influenced community responses and individual attitudes towards IPV. Education initiatives like Safe Dates in the USA have been applauded for effectively reducing the perpetration of IPV [39, 40], however the impact of similar initiatives like life-skills in Uganda is yet to be experienced.

Region was significantly associated with all forms of IPV. Particularly, women in the Northern and Western regions of Uganda had higher odds of experiencing emotional, physical and any IPV. Women in Eastern and Western Uganda were also more likely to experience sexual IPV compared to those in Central Uganda. This finding is consistent with other studies by Karamagi, Tumwine [41], Wandera, Kwagala [26] and Annan and Brier [42] where they found that structural factors, like gender inequality, devastating poverty, alcoholism and police corruption, helped sustain IPV. The social acceptance of violence as a tool to resolve conflicts in relationships among some Ugandan societies alongside weak implementation of community sanctions against IPV could explain this finding.

Witnessing parental violence was significantly associated with all the forms of IPV. Women who witnessed their fathers beat their mothers were more likely to experience all the forms of violence compared to their counterparts. Visual learning is an effective mind mapping tool that promotes a community of practice. Also, the social learning theory’s argument that perpetration and acceptance of violence are a learned behavior could help explain this finding. This learned behavior is seen among men who perpetrate violence because they witnessed their fathers’ violent actions towards their mothers and among women who accept violence because they saw their mothers being abused by their fathers. Recent studies in Uganda and other parts of the world have reported a similar trend [21, 26, 43, 44].

Women whose partners had controlling behaviors were more likely to experience all forms of IPV and similar findings have been reported in Uganda [24–26] and in the WHO multi-country study [13]. The patriarchal societies that emphasize male dominance in the family and the dowry that men pay could help explain this finding.

Duration of relationship was related with all the forms of IPV. Women in a relationship for longer durations had increased odds of experiencing violence compared to those with shorter durations. This finding contradicted Urquia, O’Campo [45] who indicated that as much as marital duration was significantly associated with IPV, it is only women with shorter marital duration who experienced more IPV than those with longer marital duration. The explanation could be that women with longer marital duration remain in abusive relationships for the sake of their children and the fear of humiliation that society bestows to those that separate or divorce, in addition to the potential difficulties in starting new relationships.

Once one’s desire to drink becomes uncontrollable, alcoholism becomes inevitable and this catalyzes the experience of violence. Similarly, the frequency of a partner being drunk was another predictor of emotional, sexual, physical and any IPV. Women whose partners were often or sometimes drunk had increased odds of experiencing IPV compared to their counterparts whose partners were never drunk. Perhaps men who perpetrate violence while drunk lose control over their behaviors or use alcohol as an excuse for their behavior. Literature that supports this finding indicated that harmful use of alcohol and/ or problem drinking was a precursor to the experience of IPV [30, 46, 47].

Age of the woman was a predictor of emotional, sexual, physical and any IPV and it had a protective effect. Women in the age bracket 25–34 years were all less likely to experience IPV compared to their counterparts in the age bracket 15–24 years. Studies in Bolivia, the United States [22], the WHO multi-country study [48] and Uganda [41, 49] support the relationship between age and IPV.

Age at first marriage was significantly associated with sexual IPV, with women who married after 18 years of age being more likely to experience sexual violence compared to those who married before the age of 18. The legal age of marriage in Uganda is 18 years old. Studies in Canada [45] and India [50] were in agreement with this study’s finding. Perhaps these women have a relatively low relationship power and cannot make certain independent decisions.

Wealth status was significantly associated with physical IPV. Our finding is consistent with the pattern reported in India [22, 51] and Uganda [24, 29] where women with higher income and those from higher socio-economic status have decreased odds of experiencing violence. However, this study also indicated that women in the poorer and middle wealth quintiles also had lower odds of experiencing physical violence. This seems to contradict other studies [13, 21] that reported otherwise.

Closely related to the above, economic empowerment was protective against sexual IPV since empowered women were less likely to experience sexual violence. Our findings are consistent with previous studies [24, 25, 52] that indicated that empowered women contribute financially to household needs, get involved in decision making and have lower odds of experiencing intimate partner violence.

The number of children (parity) a woman has was associated with emotional, physical and any IPV. Women with one or more children had increased odds of experiencing emotional, physical and any IPV. This finding is in consonance with findings in the DHS Analytical Studies [21], India [53] and Uganda [25].

Concerning the different models, the three models on emotional, sexual and physical IPV culminated into correlates specific to emotional, sexual and physical violence, respectively. However, the generalised model on any IPV lead to correlates and also knowledge that can be generally applied to new situations and environments.

### Study limitations

The key limitation was using cross-sectional data. First, we cannot ascertain causality among key variables. In addition, self-reporting of different forms of IPV is associated with social desirability biases and underreporting. Also, there are a number of other key predictors that have been established in other social settings [1, 13, 28]. Such predictors include marital unhappiness, communication issues, personality, stress, hot button issues, long standing mental health, attitudes, and provoking acts by partners, among others. Unfortunately, due to lack of data on these predictors, they were excluded from analysis. Notwithstanding the limitations, this study provides a robust estimation of the different forms of IPV among women in a relationship age 15–49 years using a nationally representative sample.

## Conclusion

Different forms of IPV among women in a relationship had various predictors. The cross cutting correlates for the individual forms of IPV (emotional, sexual and physical) included age, region, witnessing parental violence, partner’s controlling behaviors, duration of the relationship, and frequency of male partners being drunk. Parity was a correlate of both emotional and physical IPV. The unique predictors of sexual IPV were economic empowerment and age at first marriage. The overall (generalised) model on any IPV was associated with age, region, wealth status, partners’ controlling behaviors, frequency of partner being drunk, witnessing parental violence, duration of relationship and parity.

In a bid to lessen IPV, we recommend a qualitative research approach to help gain a deeper understanding of the region specific issues (structural factors) that underpin regional IPV variations. Also, an investigation into the willingness of men to partake in Adult Education and Lifelong Learning opportunities as a formula to reduce controlling behaviors, alcohol consumption and the number of women they marry is needed. More resources and program interventions should be mobilized by policy makers, public health experts and researchers towards the problem of IPV. Furthermore, more data is required in this area to set up evidence-based strategies that respond to and prevent IPV.

## Data Availability

The datasets generated and/ or analysed during the study are available in the DHS Program repository, available at www.dhsprogram.com

## Abbreviations

GBV: Gender Based Violence
IPV: Intimate Partner Violence
UDHS: Uganda Demographic and Health Survey
